# Nanoparticle-assisted optical tethering of endosomes reveals the cooperative function of dyneins in retrograde axonal transport

**DOI:** 10.1038/srep18059

**Published:** 2015-12-10

**Authors:** Praveen D. Chowdary, Daphne L. Che, Luke Kaplan, Ou Chen, Kanyi Pu, Moungi Bawendi, Bianxiao Cui

**Affiliations:** 1 Department of Chemistry, Stanford University, 380 Roth Way, Stanford, CA 94305, USA; 2 Department of Chemistry, Massachussets Institute of Technology, 77 Massachussets Ave, Cambridge, MA 02139, USA; 3 School of Chemical and Biomedical Engineering, Nanyang Technological University, 62 Nanyang Drive, N1.3, B2-05, Singapore 637459

## Abstract

Dynein-dependent transport of organelles from the axon terminals to the cell bodies is essential to the survival and function of neurons. However, quantitative knowledge of dyneins on axonal organelles and their collective function during this long-distance transport is lacking because current technologies to do such measurements are not applicable to neurons. Here, we report a new method termed nanoparticle-assisted optical tethering of endosomes (NOTE) that made it possible to study the cooperative mechanics of dyneins on retrograde axonal endosomes in live neurons. In this method, the opposing force from an elastic tether causes the endosomes to gradually stall under load and detach with a recoil velocity proportional to the dynein forces. These recoil velocities reveal that the axonal endosomes, despite their small size, can recruit up to 7 dyneins that function as independent mechanical units stochastically sharing load, which is vital for robust retrograde axonal transport. This study shows that NOTE, which relies on controlled generation of reactive oxygen species, is a viable method to manipulate small cellular cargos that are beyond the reach of current technology.

Cytoplasmic dynein drives the retrograde transport of various organelles including vesicles, endosomes, autophagosomes, lysosomes, mitochondria etc., from the axon terminals to the cell bodies in neurons^1^,^2^,^3^,^4^,^5^. This long distance retrograde axonal transport mediates many signaling, clearance, and degradation mechanisms that are fundamental to the neuronal structure, function and survival^3^,^4^. The extreme length of axons, ranging from millimeters up to a meter, is many orders of magnitude higher than the average runlength (<1.5 μm) of a single dynein *in vitro*^6^. Further, the viscoelastic forces and hydrodynamic drag^7^,^8^ within the narrow caliber axons can potentially stall a single dynein which is a weak motor with modest stall force of ~1.1 pN^9^,^10^,^11^. Yet large numbers of retrograde cargos are robustly transported across the entire axon at an average speed of 1–2 um/s^12^,^13^,^14^,^15^. Indeed, such fast transport of retrograde endosomes carrying growth factors is critical for the balance between survival and apoptotic signaling pathways in neurons^16^. The robustness of long-distance retrograde transport can be attributed to the collective mechanics of multiple dyneins^10^ and/or intracellular regulation of dynein properties^17^. Elucidating the mechanics of retrograde cargo transport can lead to fundamental insights on its vulnerability to defects in dynein function^13^,^18^ and adverse conditions like traffic jams implicated in numerous neurodegenerative disorders^3^,^19^,^20^,^21^,^22^,^23^.

The collective function of multiple dyneins is shown to enhance cargo runlengths *in vitro*^9^,^24^ and can generate forces up to 20 pN as shown for a few large cargos (0.7–1 μm diameter) in cells^9^,^10^. However, the stoichiometry of active dyneins and their cooperative function on retrograde axonal cargos overcoming tremendous distances are not understood. Immunoblotting and immunostaining approaches^25^,^26^ cannot probe the activity of motors and as such there is no quantitative data yet on retrograde axonal cargos. Stochastic model simulations of retrograde endosome motility in axons suggest that ~5–6 dyneins are involved but this needs experimental substantiation^27^. The only existing method to probe the number of active motors and their collective mechanics on cellular cargo is by measuring the forces acting on the cargo using calibrated optical traps^10^,^11^,^28^,^29^. However, these approaches are limited to a few large cellular cargos, such as lipid droplets and latex bead phagosomes that can be manipulated by optical forces. Retrograde axonal cargos, particularly vesicles and endosomes ~50–250 nm in size^12^,^25^, are not amenable to any of the existing force measurement techniques.

In this paper, we report a novel phenomenon termed nanoparticle-assisted optical tethering of endosomes (NOTE) that made it possible to study the collective function of dyneins on retrograde axonal endosomes in live neurons. We have previously developed a microfluidic platform that allows us to visualize retrograde axonal endosome transport using ligand-bound quantum dots and oblique illumination imaging in microfluidic neuron cultures^4^,^27^. The ligand can be growth factors (NGF, BDNF, etc.)^4^, viruses^30^,^31^, toxins^32^, and lectins^1^,^33^ etc. Briefly, the binding of a quantum dot labeled ligand to its membrane receptor at the axon terminal leads to the internalization of the quantum dot-ligand-receptor complex into an endosome, which is retrogradely transported across the axons by dyneins (Fig. 1a). In this work, we studied the retrograde endosome transport of wheat germ agglutinin (WGA)-coated quantum dots, magnetic nanoparticles, and polymer nanoparticles. Intriguingly, we found that widefield laser illumination of endosomes carrying fluorescent nanoparticles led to the endosomes being stochastically tethered in axons. The tether essentially acts like an elastic spring, with stiffness <0.01 pN/nm, opposing the motion of dyneins driving the endosomes. The motility of tethered endosomes, recorded at 150 fps and <25 nm spatial resolution, reveals the cooperative mechanics of up to 7 dyneins stochastically sharing the load on endosomes. This highlights that the collective function of dyneins is generic over a wide range of cargo sizes and is vital for the long-distance retrograde axonal transport in neurons. Our results suggest that photogeneration of reactive oxygen species (ROS) plays a critical role in NOTE. We also show that controlled ROS generation by nanoparticle design can be a viable technique for quantitative studies on intracellular cargo transport.

## Results

### Retrograde axonal transport of WGA-endosomes in microfluidic DRG neuron cultures

**Figure 1:**
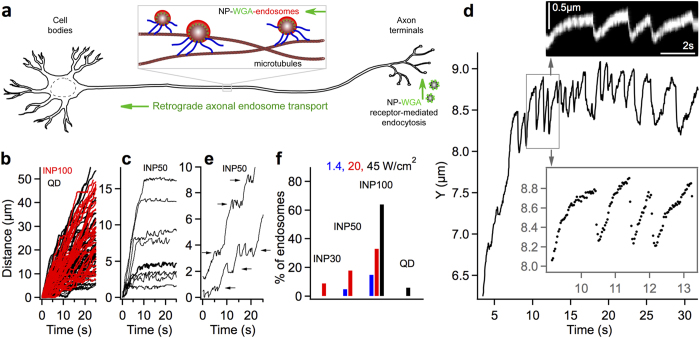
Nanoparticle assisted optical tethering of endosomes in axons. (**a**) Schematic of the retrograde axonal transport of nanoparticle-WGA endosomes from axon terminals to the cell bodies in neurons. (**b**) Unperturbed retrograde transport trajectories of QD-endosomes (black, 45 W/cm^2^, 32fps) and INP100-endosomes (red/gray, 1.4 W/cm^2^, 10fps). (**c**) Retrograde INP50-endosomes (19 W/cm^2^, 32fps) stochastically becoming stationary within the imaging field of view. (**d**) Gradual stalling and fast reversals (jumps) exhibited by an affected INP-endosome. Inset zooms in on a few jumps with the corresponding kymograph is also shown. (**e**) Laser-affected INP50-endosomes exhibiting jumps at different locations along the axon. (**f**) Percentage of laser-affected endosomes, for different nanoparticles at varying laser powers.

If the viscous load on a motor-driven cargo is high enough to affect the stepping speed of motors, the cargo velocity would depend on the number of motors actively sharing the load. Indeed, multimodal velocity distributions of cellular cargos under high cytosolic viscous load have previously been interpreted in terms of the number of motors active on the cargo^34^,^35^. So we first asked if the velocity of retrograde endosomes in axons reflects the number of dyneins on the endosomes. To this end, we analyzed the retrograde axonal transport of Alexa-WGA (~2 nm), WGA-coated quantum dots (QDs, ~15 nm) and WGA-coated fluorescent iron oxide nanoparticles of different sizes (INPs, 30, 50, 100 nm) (Fig. 1a). WGA is an established retrograde tracer^1^,^36^ with a high density of membrane receptors that is shown to promote robust endocytosis and transport of nanoparticles in cells^37^. For comparison, we also analyzed the motility of retrograde endosomes stained by a small lipophilic dye (DiI, molecular weight = 933.9 g) that is selectively applied to the axon terminals.

We tracked the retrograde axonal transport of endosomes in real time using oblique illumination imaging in microfluidic DRG neuron cultures as described earlier^4^,^27^. The imaging was carried out in axonal segments far from (hundreds of microns) the terminals and cell bodies, following distal incubation of Alexa-WGA (0.5 nM), QD-WGA (0.5 nM), INP-WGA (1–2 nM), or DiI (2μM). We obtained the endosome trajectories from the time-lapse movies using particle-tracking approaches described in the Supplementary Information. The endosome motion is almost unidirectional (retrograde) and highly processive (Fig. 1b). Most endosomes traversed the imaging field of view (~85 μm) with no indication of detachment from the microtubules or diffusion within axons (Supplementary Movies S1, S2). The retrograde transport velocities of Alexa-WGA (Mean ± SD, 1.88 ± 1.1 μm/s), QD-WGA (1.90 ± 1.2 μm/s) and INP-WGA (1.8 ± 0.7 μm/s) endosomes are comparable and the endosome run velocity distribution ranged from 0.25 to as high as 5 μm/s (Supplementary Fig. S4). The mean retrograde endosome velocity is comparable to the unloaded dynein velocity (~0.6–1 μm/s *in vitro* at 24 °C^6^,^38^), considering the twofold increase in endosome velocities going from 24 to 37 °C^27^,^39^. This indicates that the endosome transport in axons is in a low viscous load regime within the size range of Alexa-, QD-, and INP-WGA endosomes^40^. This is consistent with the low effective viscosity ~0.1 *Ns/m*^*2*^ experimentally estimated for endosomes in axons^27^. Therefore, endosome velocity is not a viable measure for the number of dyneins active on the retrograde endosomes.

### Nanoparticle-assisted optical tethering of endosomes (NOTE) in axons

At low imaging illumination power (1.4 W/cm^2^, 561 nm) the INP-endosome motility is similar to that of QD-endosomes (Fig. 1b). However, the transport of INP-endosomes exhibited an intriguing dependence on the laser power. Specifically, around a threshold power of 10–20 W/cm^2^ many of the smoothly moving INP-endosomes stochastically became stationary within the imaging field of view (Fig. 1c). Before becoming stationary, the affected INP-endosomes exhibited a repeated pattern of gradual stalls followed by fast reversals in motion, referred to as ‘endosome jumps’ in this paper (Fig. 1d, Supplementary Movie S3). The jumping speed ranged as high as 100 μm/s, which is substantially faster than molecular motor driven motion. In most cases the endosome jumps were confined to a narrow region of <2μm within the site of the first jump along the axon (Fig. 1c). However, some of the laser affected INP-endosomes resumed transport after a few jumps and exhibited intermittent jumps at different locations along the axon (Fig. 1e). Occasionally, the affected INP-endosomes resumed normal retrograde motion after a few jumps and left the imaging field of view. The fraction of INP-endosomes that were perturbed by laser illumination (exhibiting >3 jumps or suddenly becoming stationary within 30s of imaging) increased with the laser power and the size of INP (Fig. 1f). For INP100 at 45 W/cm^2^, a majority of endosomes (~64%) exhibited jumps and/or became stationary upon entering the illumination field. We note that this phenomenon of laser-induced endosome jumps/stalling is striking with fluorescent INPs. QD-endosomes and DiI-stained retrograde endosomes in axons are insensitive to the laser power in this range and rarely (<6% at 45 W/cm^2^) exhibited such jump pattern (Fig. 1f, Supplementary Fig. S5, Supplementary Movies S4 and S5).

**Figure 2:**
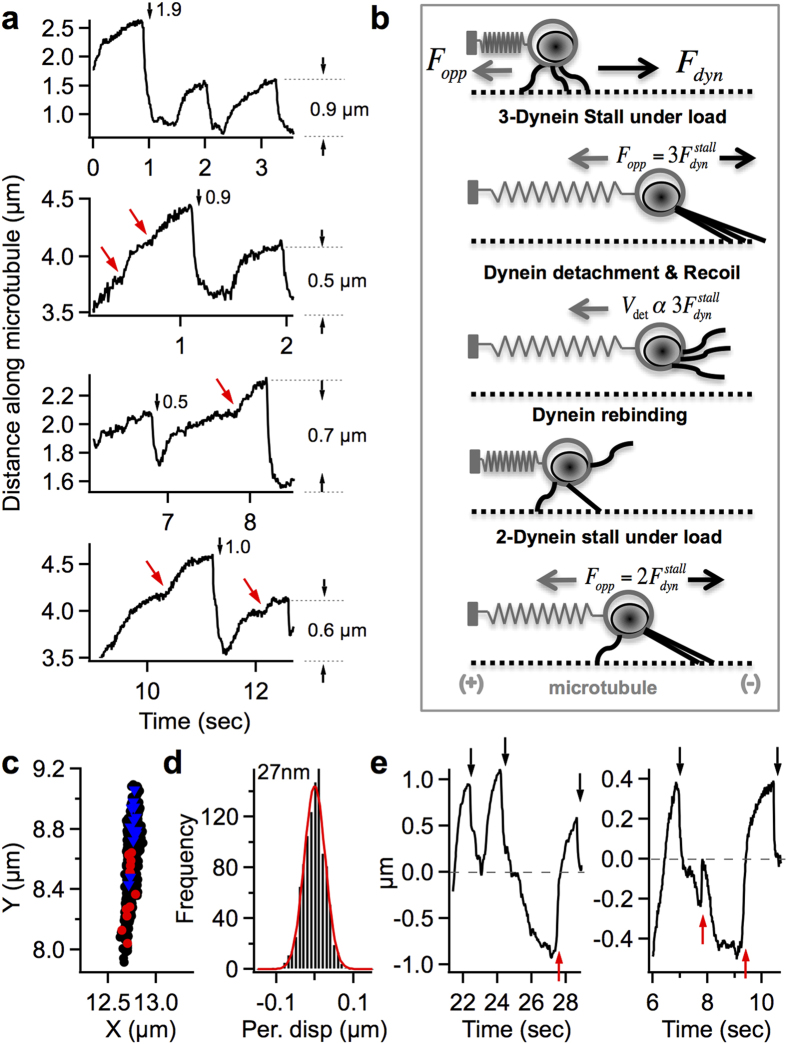
Stochastic load-sharing model and the nature of *F*_*opp*_ in NOTE. (**a**) INP-endosome jumps of different sizes labeled with the approximate jump sizes (black arrows and dotted lines). The typical shoulders in stall profiles (red/gray arrows) indicate stochastic load-sharing among endosome-bound dyneins. (**b**) Stochastic load sharing model for tethered endosome motion. The endosome jumps are explained in terms of the stalling and detachment of the leading dyneins under the elastic opposing force (*F*_*opp*_) of stretched tether. (**c**) Tracked positions of the INP-endosome in Fig. 1e reflecting the underlying microtubular track. Endosome positions just before dynein detachment (blue/triangles) and 125 ms after detachment (red/circles) show that the jumps are parallel to the microtubular track. (**d**) Distribution of INP-endosome displacement perpendicular to microtubule. (**e**) Occasional reversed motion seen after jumps (black arrows), resulted in a stall/jump in the opposite direction (red/gray arrows).

The repeated stalls and fast reversals in Fig. 1d (endosomes jumps) closely resemble the movements of dynein driven cargo under load in optical traps, both *in vitro* and in cells^10^. Figure 2a shows the typical profiles of INP-endosome jumps of varying sizes (approximately estimated as shown by the dotted lines). The gradual stalling prior to a jump and the characteristic shoulders seen in the stall profiles are remarkably similar to those of multiple dyneins sharing load stochastically^10^. Our data can be explained by considering that the laser-affected INP-endosomes are docked by an elastic tether within axons (Fig. 2b). The dyneins pulling the tethered INP-endosome are slowed down by the elastic tether force (*F*_*opp*_) opposing the endosome motion as shown in Fig. 2b. The endosome gradually stalls as the tether is stretched and *F*_*opp*_ reaches the stall force of the leading dyneins. Once the dyneins at stall detach from the microtubule, the endosome recoils back under the influence of *F*_*opp*_ resulting in the jump. The jump size depends on the number of dyneins collectively stretching the tether.

Several features of the endosome jumps indicate that *F*_*opp*_ is a microtubule-centered force. First, the tracked positions of INP-endosomes during a series of jumps show that the endosome recoil after dynein-detachment is parallel to the microtubular track, indicating that *F*_*opp*_ acts along the microtubule (Fig. 2c, Supplementary Fig. S5). A randomly positioned tether in the axon is expected to cause the recoil at an angle to the microtubule. Second, the endosome displacement perpendicular to the microtubule during a series of jumps is ~20–30 nm which is the localization accuracy of our system (Fig. 2d, Supplementary Fig. S5). This suggests that the endosome motion is confined to the same microtubule during the jumps. Third, INP-endosomes occasionally reversed direction (by anterograde kinesin motors) following a jump and exhibited a similar stall/detachment behavior in the opposite direction (Fig. 2e, opposite jumps indicated by red arrows). These observations suggest that the opposing force *F*_*opp*_ comes from a microtubule-centric tether, which holds the endosome from moving in both retrograde and anterograde directions. More importantly, this indicates that *F*_*opp*_ at stall is in perfect counterbalance with the cumulative force exerted by dyneins on the retrograde endosomes. Therefore, the endosome recoil velocity upon dynein detachment and the jump size, which are proportional to *F*_*opp*_, provide quantitative insights on the leading-dyneins.

We refer to this phenomenon of laser-induced INP-endosome jumps as nanoparticle-assisted optical tethering of endosomes (NOTE). The endosome jumps caused by NOTE make it possible to study the cooperativity of dyneins on axonal endosomes, which are not amenable to force manipulation as such. In what follows we extract quantitative information about the collective function of dyneins on axonal endosomes from the measured endosome jumps.

### Load-induced motor detachment model for the tethered endosome jumps

We considered a simple model for endosome jumps. The endosomes are modeled as hard spheres with minimal deformation during jumps. This is confirmed by the analysis of shape deformation in DiI-stained retrograde endosomes during jumps (Supplementary Fig. S6). The elastic tether force in NOTE serves as an external load on the dyneins pulling the tethered endosomes. The stochastic motion of motor-driven tethered endosomes is governed by the Langevin equation (Equation (1)) including the motor force, frictional force, Brownian forces and the tether force on the endosome^41^,^42^.


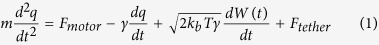


where *m* is the mass of the endosome, *γ* is the effective friction coefficient of the endosome, *W(t)* represents the Brownian forces and *F*_*tether*_ = *−k(q−q*_*0*_) is the force exerted by the stretched tether, modeled as an elastic spring of stiffness ‘*k’*. Given the mean endosome size *d*~100 nm and experimentally estimated axonal viscosity^27^ *η* ~ 0.1 *Ns/m*^*2*^, the inertial motion timescales of the endosome and axoplasm, given by *τ*_*p*_ = *m/γ* and *τ*_*f*_ = *d*^*2*^*ρ*_*f *_*/4η* respectively^42^, are <30ps. Within the time resolution of our imaging (>6.75 ms), we can safely neglect the fast inertial motions. The endosome motion can therefore be described by the Langevin equation without inertia^41^ that is further reduced to Equation (2) upon ignoring the Brownian term, which is at a much faster time scale than the endosome motion under the influence of motors/tether.


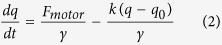


**Figure 3:**
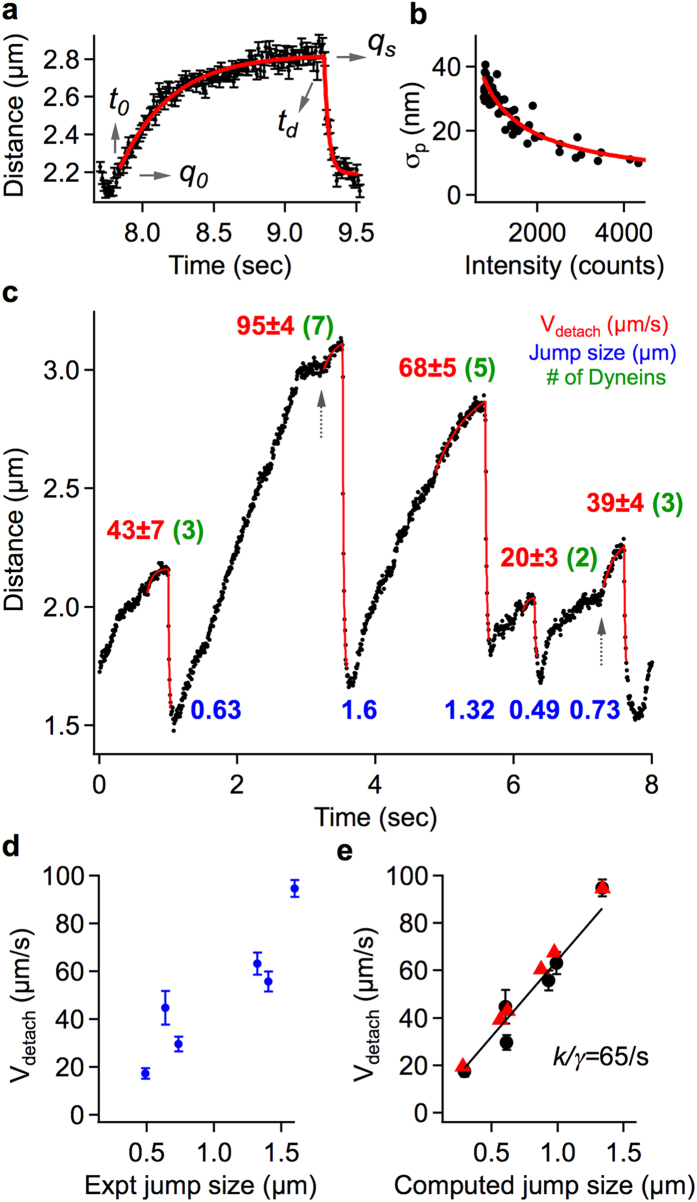
Detachment velocities from fitting the endosome jumps. (**a**) Global fitting (red) of pre- and post-detachment curves to estimate the detachment time and position (*q*_*s*_*, t*_*d*_), detachment velocity *V*_*detach*_, and damping constant *k/γ*, from an endosome jump. (**b**) Power law calibration (red) of position uncertainties (black, standard deviation over 10s of tracking of coverslip bound INPs as a function of fluorescence intensity) used in model fitting. (**c**) Retrograde INP100-endosome trajectory exhibiting a range of jump sizes and multiple-dynein stall profiles (dotted arrows) at 45 W/cm^2^, 150fps. Experimental jump sizes (near multiples of 0.23 μm) are labeled in blue and the detachment velocities *V*_*detach*_ (near multiples of 13 μm/s) are labeled in red. (**d**) Approximate linearity of *V*_*detach*_ with experimental jump size. e) *V*_*detach*_ obtained with *k/γ* as a fitting parameter (black) or fixed at 65/s (red) plotted vs the computed jump size.

Equations (3), (4) represent the solutions of Equation (2) before and after motor detachment under load respectively (fitted red curves in Fig. 3a, Details in Supplementary Information).


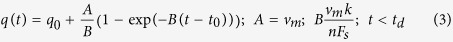



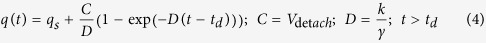


Where, *v*_*m*_ is the unloaded velocity of the endosome in Stokes regime, *nF*_*s*_ is the stall force of *‘n’* dyneins driving the endosome, *V*_*detach*_ is the instantaneous recoil velocity of the endosome upon lead dynein detachment, and *k/γ* is the damping constant of endosome recoil. The Brownian term ignored in Equation (2) essentially adds a noise component to the endosome positions in Equations (3), (4). Figure 3b shows the localization uncertainties used in fitting the experimental data to this model. The main parameters obtained by fitting the endosome jumps to our model are *V*_*detach*_ and *k/γ*. Since the inertial endosome motion is instantaneous on the timescale of imaging, *V*_*detach*_ is given by *F*^*stall*^*/γ* in the Stokes regime. *F*^*stall*^ is the force acting on the stalled endosome, which equals the cumulative stall force of the leading dyneins. The distribution of *V*_*detach*_ obtained over a large population of retrograde endosome jumps contains crucial information about the number of dyneins and their collective function on endosomes as elaborated below.

### Fast imaging of INP100-endosome jumps to extract instantaneous detachment velocities

The endosome recoil after lead dynein detachment on average lasted ~150 ms before the dynein driven motion resumed for the next stall/jump. However, the exponential recoil is often interrupted within 40–60 ms possibly by the presence of a lagging bound dynein, or by fast rebinding of detached dyneins, or collisions with cytoskeletal features. Therefore, it was important to accurately capture the initial 40 ms of endosome recoil, which entailed an imaging rate >100 fps. Using highly fluorescent INP100 we could image a field of 18 × 90 μm^2^ at 150fps with localization accuracy <25 nm (with 45 W/cm^2^ laser power).

Figure 3c shows the trajectory of a tethered retrograde INP100-endosome, imaged at 150fps, exhibiting a series of stalls and detachments (Supplementary Movie S6, Supplementary Fig. S2). There are several interesting features that reflect the cooperative activity of multiple dyneins on this endosome, which are consistent with our model. First, the experimental jump sizes (estimated as in Fig. 2a) are near-multiples of 0.23 μm and the characteristic stall profiles indicate stochastic load sharing among multiple dyneins^10^,^43^. Second, the instant detachment velocities *V*_*detach*_, obtained by fitting each jump, are approximately linearly related to the experimental jump size over a wide stretching range (0.2–1.4 um) (Fig. 3d). This suggests that the tether force is elastic and the force is appropriately modeled as *–k(q-q*_*0*_). We note that the experimental jump size *(q*_*s*_*-q*_*0*_) is approximate since the tether location *q*_*0*_ is not precisely known. This is because A) the velocity change at low load (mildly stretched tether) is not always obvious due to the load sharing by dyneins, and B) the endosome recoil is not always complete due to fast dynein rebinding. However, from the fitted parameters *V*_*detach*_ and *k/γ* for each jump, we can compute the jump size using the relation *F*^*stall*^ = *γV*_*detach*_ = *k(q*_*s*_*-q*_*0*_). The computed jump size shows good linearity with *V*_*detach*_, whose slope gives a constant *k/γ* = 65/s for this endosome (black dots in Fig. 3e). Since the effective friction coefficient *γ* is constant for a given endosome, this implies that the tether stiffness *k* is constant and not varying from jump to jump for this endosome.

The dispersion seen in the fitted *k/γ* values for individual jumps comes from the standard errors in fitting the jumps. So we fixed *k/γ* to 65/s and fit all the jumps for more accurate estimates of *V*_*detach*_ (red triangles in Fig. 3e). Though the number of jumps is limited, the detachment velocities could be interpreted as integral multiples of ~13 μm/s (red numbers in Fig. 3c). The green numbers in Fig. 3c are estimated leading dyneins at each jump. For a ~100 nm endosome within an effective viscosity^27^ *η* ~ 0.1 *Ns/m*^*2*^, the 13 μm/s velocity corresponds to a force of 1.2 pN in Stokes regime which is comparable to the 1.1–1.25 pN stall force of a single dynein^10^,^11^,^44^. The trajectory in Fig. 3c suggests that up to 7 dyneins are active on this endosome. Assuming that the smallest jump size of 0.23 μm is a single dynein stall at 1.1pN, the tether stiffness *k* is estimated to be ~0.005 pN/nm.

### Discrete detachment velocity distribution of retrograde endosome population

**Figure 4:**
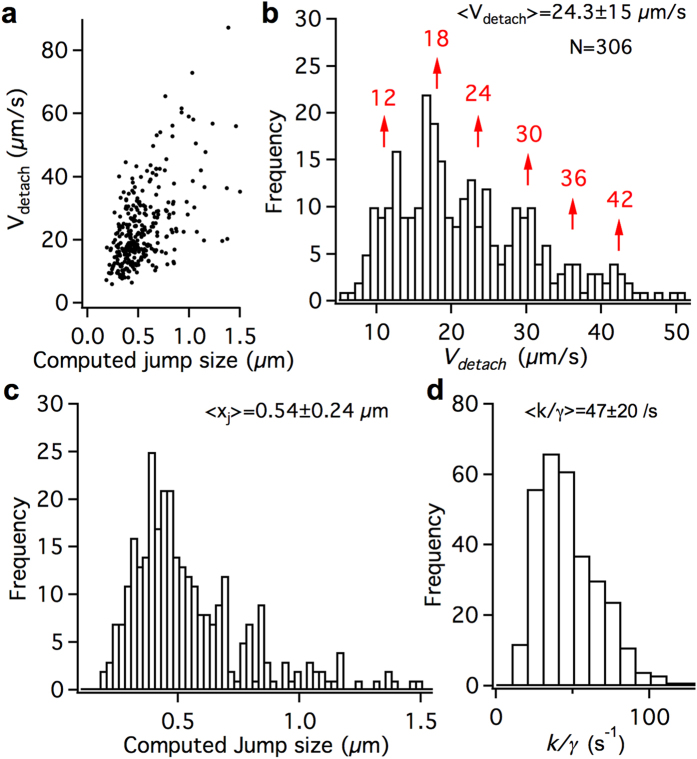
Parameter distributions for retrograde INP100-endosomes. (**a**) Linear correlation of *V*_*detach*_ with the computed jump size for the retrograde INP100-endosomes (45 W/cm^2^, 150fps). The dispersion from linearity results from the variation in *k/γ* among the endosomes and the standard errors (SE) in fitting. Only jumps with <15% SE in *V*_*detach*_ (306 out of 961 jumps) are considered here. (**b**) Distribution of *V*_*detach*_ exhibits discrete peaks that are near multiples of ~6 μm/s (**c**) Distribution of the computed jump size. (**d**) Distribution of *k/γ*. The mean ± sd are shown for the distributions in b, and d.

We then analyzed the jumps for a large population of retrograde INP100-endosomes with an average of 3 jumps per endosome (Supplementary Fig. S3). From a total of 961 jumps fit to our model, we only selected the 306 jumps that gave <15% standard error in the fit *V*_*detach*_ value. Figure 4a shows that the detachment velocities (*V*_*detach*_) obtained by fitting are approximately linearly correlated with the computed jump size. Figure 4b shows the distribution of *V*_*detach*_, with a mean of 24.3 μm/s and extending beyond 50 μm/s. The distribution shows multiple peaks with a multiplicity of ~6 μm/s, which can be interpreted as the detachment velocity after a single dynein stall event of the average sized INP100-endosome. Assuming an average INP100-endosome size of ~150 nm and an effective viscosity *η* ~ 0.1–0.2 *Ns/m*^*2*^, the 6 μm/s velocity corresponds to a single dynein stall force of 0.89–1.8 pN which is comparable to the 1.1–1.25 pN unitary stall force of dynein^10^,^11^,^44^. The largest discernable peak at 42 μm/s suggests that up to 7 dyneins can be active on the endosomes. Notably, the mean *V*_*detach*_ of ~24 μm/s indicates that on average ~4 dyneins cooperatively share the load on endosomes. We do not see a prominent peak at 6 μm/s. It is likely that the single dynein stall events are less frequent since the molecular adaptations within dynein are highly conducive to cooperation and load sharing^10^. Further, some of the single dynein jumps may have been missed during data fitting since it was difficult to distinguish 6 μm/s jumps from intermittent anterograde direction reversals (driven by kinesin motors), which can have comparable velocities at 37 °C^27^.

Figure 4c shows the computed jump size distribution, with a mean of 0.54 μm and ranging up to 1.5 μm. The discrete peak structure is not quite obvious in the jump size distribution, possibly due to variation in tether stiffness *k* among the endosome jumps. If the average jump size (~0.54 μm) corresponds to 4 dyneins (4.4 pN), the average tether stiffness k can be estimated to be ~0.008 pN/nm. Figure 4d shows the distribution of the damping constant *k/γ*, with a mean of 47/s and ranging up to 110/s. The friction coefficient *γ* (proportional to endosome size) varies from one endosome to another within the distribution. However, the discrete multiplicity of *V*_*detach*_ distribution indicates that the variation in *γ* (i.e. endosome size) is not significant. It is plausible that the large INP100 particles may have narrowed the size distribution of endosomes, shown to be 50–150 nm for QDs^12^, by truncating the smallest sizes.

### Stall duration as an independent metric for estimating the number of dyneins sharing load

**Figure 5:**
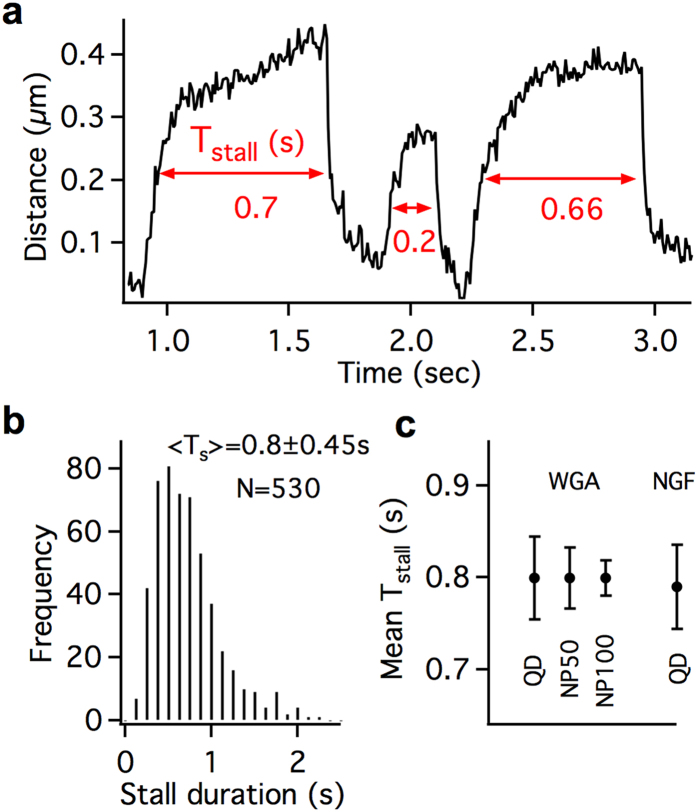
Stall duration of endosomes prior to detachment. (**a**) *T*_*stall*_ duration shown for a few INP100-endosome jumps. (**b**) Distribution of the stall duration *T*_*stall*_ for INP100-endosomes (**c**) Mean *T*_*stall*_ for different nanoparticle/ligand endosomes.

Another independent metric related to the number of cooperating dyneins on endosomes is the stability under load represented by the stall duration *T*_*stall*_ before detachment^10^,^45^. *T*_*stall*_ is defined as the time spent above half the cumulative stall force prior to the dynein detachment within a jump (Fig. 5a). The concave force-velocity relationship, adaptable step-size and catch-bond detachment kinetics of dynein aid in efficient load-sharing and also enhance the tenacity of a team of dyneins against detachment under load. Recently calibrated force measurements showed that *T*_*stall*_ grows linearly with the number of cooperating dyneins^10^.

While the detachment velocity analysis relies on accurate post-detachment endosome recoil profiles, *T*_*stall*_ can be determined from the pre-detachment stall profiles. Only a subset of the INP100-endosome jumps (N = 530 out of 961) exhibited the full range of pre-detachment stall profile (Fig. 5a) for which *T*_*stall*_ could be estimated. The mean *T*_*stall*_ for these jumps is 0.8 ± 0.44s (Fig. 5b), which approximately corresponds to an average force of ~5 pN according to calibrated force data in cells^10^. This corroborates our above conclusion based on *V*_*detach*_ that on average ~4 dyneins (1.1 pN stall force) are cooperatively sharing the load on the tethered INP-endosomes. Further, this shows that *T*_*stall*_ can be a viable metric to estimate the number of dyneins sharing load, irrespective of the cargo size.

Interestingly, we do not see any differences among the mean *T*_*stall*_ between QD-WGA (0.8 ± 0.45s, N = 99), INP50-WGA (0.8 ± 0.38s, N = 130) and INP100-WGA (0.8 ± 0.44s, N = 530) endosomes. This indicates that variation in the average number of leading-dyneins on these endosomes is not significant. Further, the mean stall duration of WGA-endosomes is comparable to that of QD-NGF-endosomes (0.79 ± 0.36s, N = 62)^27^, which are 50–150 nm in size^12^. This suggests that the WGA-endosomes are within a comparable size range to NGF-endosomes. Therefore, these analyses of *V*_*detach*_ and *T*_*stall*_ reveal the cooperative function of ~4 leading dyneins on axonal endosomes as small as ~100–200 nm.

### The role of reactive oxygen species (ROS) in NOTE

We hypothesize that the laser illumination induces local production of reactive oxygen species (ROS) on INP-endosomes that makes them susceptible to being tethered to a microtubule-based structure (the elastic tether). It is known that the nanoparticle surface can catalyze ROS generation under laser illumination^46^,^47^. A recent study shows that axonal transport is particularly sensitive to ROS^48^. Further, iron oxide assisted Fenton reaction can generate the highly reactive hydroxyl radical^49^,^50^. In order to analyze the role of ROS in causing the endosome jumps, we performed two complementary tests.

**Figure 6:**
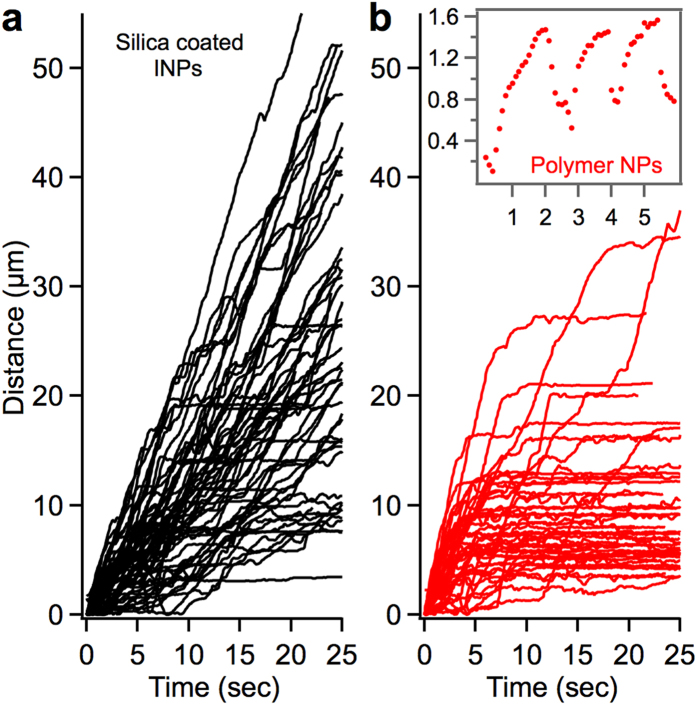
Retrograde transport of silica-coated iron oxide nanoparticles and polymer nanoparticles. (**a**) Robust retrograde transport trajectories of silica-coated INP endosomes (black, 45 W/cm^2^, 10fps, 561 nm). (**b**) Retrograde trajectories of polymer nanoparticle endosomes, stalling within seconds of entering the field of view (red/gray, 15 W/cm^2^, 10fps, 488 nm). Inset shows the endosome jumps before the endosome became stationary under the laser illumination.

Firstly, recent work showed that silica-coated INPs had reduced oxidative stress in cells^51^. So, we asked if silica passivation of INPs could reduce the endosome jump behavior. To this end, we synthesized INPs (~160 nm size) with an outer shell of QDs, passivated by a 10 nm silica layer as reported earlier^52^ (Supplementary Fig. S1). Indeed, we found that the endosomes containing the silica-coated INPs exhibited substantially reduced number of jumps (<9% at 45 W/cm^2^, Fig. 6a) compared to the endosomes with uncoated INPs (64% at 45 W/cm^2^). We observed robust retrograde transport of the silica-coated particles through the imaging field of view even at 114 W/cm^2^ power (Supplementary Movie S7). This indicates that the INP surface plays a critical role in NOTE.

Secondly, we asked if fluorophore generated ROS (without iron oxide) can induce the endosome jumps. To this end, we synthesized highly fluorescent polymer nanoparticles made of PFBT, which is a known source of photogenerated singlet oxygen, as described earlier^53^,^54^,^55^. These particles are ~30 nm in size and exhibit intense fluorescence in the range of 500–700 nm (Supplementary Fig. S1). The polymer nanoparticles are coated with WGA and are applied to the axon terminals like the QDs and INPs. Interestingly, endosomes containing the polymer nanoparticles exhibited a high sensitivity to laser illumination. Nearly 90% of these endosomes (N = 210) exhibited jumps and/or stalled within seconds of entering the laser illumination area (at 15 W/cm^2^ of 488 nm) (Fig. 6b, Supplementary Movie S8). On the other hand, QD-endosomes transported robustly under these imaging conditions. This result suggests that controlled ROS generation by the nanoparticles within endosomes can be a viable technique for tethering the endosomes and studying the mechanics of motors. However, the specific nature of ROS (hydroxyl radicals, singlet oxygen, etc), the downstream reactivity of ROS and the biochemical nature of the tether in NOTE are yet to be established.

## Discussion

Currently, there is no technology to probe the collective mechanics of dyneins on retrograde axonal cargos in live neurons. In particular, axonal endosomes sized < 250 nm^12^,^25^ are a technical challenge for force manipulation. In this work, we took advantage of a novel phenomenon termed NOTE to reveal the collective function of dyneins on retrograde endosomes in axons. We show that, despite their small size, axonal endosomes can recruit multiple dyneins that work cooperatively by stochastic load sharing. Our results indicate that the endosomes can recruit up to 7 active dyneins and on average ~4 leading dyneins share the load. This number we report specifically for retrograde endosomes is higher than the average number (1–5 dyneins) observed by immunoblotting of purified neuronal vesicles^25^ and immunostaining of prion protein vesicles in axons^26^. In comparison, latex-bead phagosomes (>750 nm size) have ~7–9 leading dyneins sharing load and *>*12 active dyneins on average^10^. Our results, in conjunction with earlier studies^10^,^45^, show that the collective function of dyneins is generic over a wide range of cargo sizes and is fundamental for the robust long-distance retrograde axonal transport. It is quite likely that the cooperativity of dyneins seen on endosomes also applies to other fast retrograde cargos like autophagosomes, lysosomes, vesicles etc. Though the primary focus of this paper has been the retrograde endosome transport by dyneins, NOTE is also applicable for studying the mechanics of anterograde endosome transport by kinesins and the inherent differences between the cooperative function of dynein and kinesin^10^.

NOTE is fundamentally different from optical traps, which use calibrated optical forces to hold onto motor driven cargos. In NOTE the elastic tether is biochemical in nature. We believe that the basic principle of NOTE is similar to that of chromophore assisted laser inactivation (CALI)^56^,^57^ and fluorophore assisted laser inactivation (FALI)^58^ that rely on photogenerated ROS. The fluorescent nanoparticle inside the endosome serves as a controllable and highly localized ROS source in NOTE. Elucidating the downstream reactivity of the specific ROS and the biochemical nature of the tether in NOTE could make it applicable beyond endosomes in axons. For instance, rare jumps have also been reported for melanosomes in melanophores^59^, QD-EGF-endosomes in epithelial cells^60^, and QD-NGF-endosomes in axons^27^ and DiI-stained endosomes (Supplementary Fig. S5). It is plausible that the ROS may stochastically cause an endosome-bound motor to remain irreversibly bound to the microtubule and act as a tether opposing the motion of the other motors. However, this entails that the motor-endosome linkage stiffness be <0.01 pN/nm to explain the observed range of endosome jump sizes. It had been suggested earlier that the motor-cargo adaptor stiffness in cells is ~0.05 pN/nm^61^ but much remains to be understood about the molecular basis of NOTE.

## Materials and Methods

A detailed description of the experimental methods, data processing and analysis (including nanoparticle synthesis and conjugation with WGA, oblique illumination imaging of axonal transport in microfluidic neuron cultures, extraction of endosome trajectories from time-lapse movies, theoretical model and the fitting of endosome jumps) is provided in the Supplementary Information.

## Additional Information

**How to cite this article**: Chowdary, P. D. *et al*. Nanoparticle-assisted optical tethering of endosomes reveals the cooperative function of dyneins in retrograde axonal transport. *Sci. Rep*. **5**, 18059; doi: 10.1038/srep18059 (2015).

## Supplementary Material

Supplementary Information

Supplementary Movie S1

Supplementary Movie S2

Supplementary Movie S3

Supplementary Movie S4

Supplementary Movie S5

Supplementary Movie S6

Supplementary Movie S7

Supplementary Movie S8

## References

[b1] von Bartheld C. S. Axonal transport and neuronal transcytosis of trophic factors, tracers, and pathogens. J Neurobiol 58, 295–314 (2004). 14704960 10.1002/neu.10315

[b2] Hollenbeck P. J. & Saxton W. M. The axonal transport of mitochondria. J Cell Sci 118, 5411–5419 (2005). 16306220 10.1242/jcs.02745 PMC1533994

[b3] Perlson E., Maday S., Fu M. M., Moughamian A. J. & Holzbaur E. L. Retrograde axonal transport: pathways to cell death? Trends Neurosci 33, 335–344 (2010). 20434225 10.1016/j.tins.2010.03.006 PMC2902719

[b4] Chowdary P. D., Che D. L. & Cui B. Neurotrophin Signaling via Long-Distance Axonal Transport. Annual Review of Physical Chemistry, Vol 63 63, 571–594 (2012). 10.1146/annurev-physchem-032511-143704 22404590

[b5] Maday S., Twelvetrees A. E., Moughamian A. J. & Holzbaur E. L. F. Axonal Transport: Cargo-Specific Mechanisms of Motility and Regulation. Neuron 84, 292–309 (2014). 25374356 10.1016/j.neuron.2014.10.019 PMC4269290

[b6] Ross J. L., Wallace K., Shuman H., Goldman Y. E. & Holzbaur E. L. F. Processive bidirectional motion of dynein-dynactin complexes *in vitro*. Nat Cell Biol 8, 562–570 (2006). 16715075 10.1038/ncb1421

[b7] Narayanareddy B. R. J., Vartiainen S., Hariri N., O’Dowd D. K. & Gross S. P. A Biophysical Analysis of Mitochondrial Movement: Differences Between Transport in Neuronal Cell Bodies Versus Processes. Traffic 15, 762–771 (2014). 24673933 10.1111/tra.12171 PMC4065622

[b8] Wortman J. C. . Axonal Transport: How High Microtubule Density Can Compensate for Boundary Effects in Small-Caliber Axons. Biophys J 106, 813–823 (2014). 24559984 10.1016/j.bpj.2013.12.047 PMC3944719

[b9] Mallik R., Petrov D., Lex S. A., King S. J. & Gross S. P. Building complexity: An *in vitro* study of cytoplasmic dynein with *in vivo* implications. Curr Biol 15, 2075–2085 (2005). 16332532 10.1016/j.cub.2005.10.039

[b10] Rai A. K., Rai A., Ramaiya A. J., Jha R. & Mallik R. Molecular Adaptations Allow Dynein to Generate Large Collective Forces inside Cells. Cell 152, 172–182 (2013). 23332753 10.1016/j.cell.2012.11.044

[b11] Hendricks A. G., Holzbaur E. L. F. & Goldman Y. E. Force measurements on cargoes in living cells reveal collective dynamics of microubule motors. Proc Natl Acad Sci USA 109, 18447–18452 (2013). 23091040 10.1073/pnas.1215462109 PMC3494964

[b12] Cui B. X. . One at a time, live tracking of NGF axonal transport using quantum dots. Proc Natl Acad Sci USA 104, 13666–13671 (2007). 17698956 10.1073/pnas.0706192104 PMC1959439

[b13] Ori-McKenney K. M., Xu J., Gross S. P. & Vallee R. B. A cytoplasmic dynein tail mutation impairs motor processivity. Nat Cell Biol 12, 1228–U1256 (2010). 21102439 10.1038/ncb2127 PMC3385513

[b14] Maday S., Wallace K. E. & Holzbaur E. L. F. Autophagosomes initiate distally and mature during transport toward the cell soma in primary neurons. J Cell Biol 196, 407–417 (2012). 22331844 10.1083/jcb.201106120 PMC3283992

[b15] Reis G. F. . Molecular motor function in axonal transport *in vivo* probed by genetic and computational analysis in Drosophila. Mol Biol Cell 23, 1700–1714 (2012). 22398725 10.1091/mbc.E11-11-0938 PMC3338437

[b16] Mok S.-A., Lund K. & Campenot R. B. A retrograde apoptotic signal originating in NGF-deprived distal axons of rat sympathetic neurons in compartmented cultures. Cell Res 19, 546–560 (2009). 19188931 10.1038/cr.2009.11

[b17] Kardon J. R. & Vale R. D. Regulators of the cytoplasmic dynein motor. Nat Rev Mol Cell Biol 10, 854–865 (2009). 19935668 10.1038/nrm2804 PMC3394690

[b18] Hafezparast M. . Mutations in dynein link motor neuron degeneration to defects in retrograde transport. Science 300, 808–812 (2003). 12730604 10.1126/science.1083129

[b19] Goldstein L. Molecular motors in neurodegenerative disease and signaling. FASEB J 19, A1334–A1334 (2005).

[b20] De Vos K. J., Grierson A. J., Ackerley S. & Miller C. C. Role of axonal transport in neurodegenerative diseases. Annu Rev Neurosci 31, 151–173 (2008). 18558852 10.1146/annurev.neuro.31.061307.090711

[b21] Brady S. T. & Morfini G. A. Dysferopathies: Neurodegenerative diseases characterized by alterations in fast axonal transport mechanisms. J Neurochem 104, 82–82 (2008).

[b22] Millecamps S. & Julien J. P. Axonal transport deficits and neurodegenerative diseases. Nat Rev Neurosci 14, 161–176 (2013). 23361386 10.1038/nrn3380

[b23] Encalada S. E. & Goldstein L. S. Biophysical challenges to axonal transport: motor-cargo deficiencies and neurodegeneration. Annual Review of Biophysics 43, 141–169 (2014). 10.1146/annurev-biophys-051013-022746 24702007

[b24] Klumpp S. & Lipowsky R. Cooperative cargo transport by several molecular motors. Proc Natl Acad Sci USA 102, 17284–17289 (2005). 16287974 10.1073/pnas.0507363102 PMC1283533

[b25] Hendricks A. G. . Motor Coordination via a Tug-of-War Mechanism Drives Bidirectional Vesicle Transport. Curr Biol 20, 697–702 (2010). 20399099 10.1016/j.cub.2010.02.058 PMC2908734

[b26] Encalada S. E., Szpankowski L., Xia C.-h. & Goldstein L. S. B. Stable Kinesin and Dynein Assemblies Drive the Axonal Transport of Mammalian Prion Protein Vesicles. Cell 144, 551–565 (2011). 21335237 10.1016/j.cell.2011.01.021 PMC3576050

[b27] Chowdary P. D., Che D. L., Zhang K. & Cui B. Retrograde axonal NGF transport - Motor coordination in the unidirectional motility regime. Biophys J 108, 2691–2703 (2015). 26039170 10.1016/j.bpj.2015.04.036 PMC4457490

[b28] Shubeita G. T. . Consequences of Motor Copy Number on the Intracellular Transport of Kinesin-1-Driven Lipid Droplets. Cell 135, 1098–1107 (2008). 19070579 10.1016/j.cell.2008.10.021 PMC2768369

[b29] Blehm B. H., Schroer T. A., Trybus K. M., Chemla Y. R. & Selvin P. R. *In vivo* optical trapping indicates kinesin’s stall force is reduced by dynein during intracellular transport. Proc Natl Acad Sci USA 110, 3381–3386 (2013). 23404705 10.1073/pnas.1219961110 PMC3587256

[b30] Ohka S. . Receptor-Dependent and -Independent Axonal Retrograde Transport of Poliovirus in Motor Neurons. J Virol 83, 4995–5004 (2009). 19244317 10.1128/JVI.02225-08 PMC2682071

[b31] Lancaster K. Z. & Pfeiffer J. K. Limited Trafficking of a Neurotropic Virus Through Inefficient Retrograde Axonal Transport and the Type I Interferon Response. PLoS Pathog. 6 (2010). 10.1371/journal.ppat.1000791 PMC2832671 20221252

[b32] Lalli G., Bohnert S., Deinhardt K., Verastegui C. & Schiavo G. The journey of tetanus and botulinum neurotoxins in neurons. Trends Microbiol 11, 431–437 (2003). 13678859 10.1016/s0966-842x(03)00210-5

[b33] Butowt R. & von Bartheld C. S. Connecting the dots: trafficking of neurotrophins, lectins and diverse pathogens by binding to the neurotrophin receptor p75(NTR). Eur J Neurosci 17, 673–680 (2003). 12603257 10.1046/j.1460-9568.2003.02497.x

[b34] Hill D. B., Plaza M. J., Bonin K. & Holzwarth G. Fast vesicle transport in PC12 neurites: velocities and forces. European Biophysics Journal with Biophysics Letters 33, 623–632 (2004). 15071760 10.1007/s00249-004-0403-6

[b35] Levi V., Serpinskaya A. S., Gratton E. & Gelfand V. Organelle transport along microtubules in Xenopus melanophores: Evidence for cooperation between multiple motors. Biophys J 90, 318–327 (2006). 16214870 10.1529/biophysj.105.067843 PMC1367030

[b36] Liu S.-L. . Visualizing the endocytic and exocytic processes of wheat germ agglutinin by quantum dot-based single-particle tracking. Biomaterials 32, 7616–7624 (2011). 21764443 10.1016/j.biomaterials.2011.06.046

[b37] Gao X. L. . Quantum dots for tracking cellular transport of lectin-functionalized nanoparticles. Biochem Biophys Res Commun 377, 35–40 (2008). 18823949 10.1016/j.bbrc.2008.09.077

[b38] King S. J. & Schroer T. A. Dynactin increases the processivity of the cytoplasmic dynein motor. Nat Cell Biol 2, 20–24 (2000). 10620802 10.1038/71338

[b39] Zhang K. . Single-molecule imaging of NGF axonal transport in microfluidic devices. Lab Chip 10 (2010). 10.1039/c003385e PMC2935512 20623041

[b40] Martinez J. E., Vershinin M. D., Shubeita G. T. & Gross S. P. On the use of *in vivo* cargo velocity as a biophysical marker. Biochem Biophys Res Commun 353, 835–840 (2007). 17196170 10.1016/j.bbrc.2006.12.120 PMC2889695

[b41] Wang H. Motor potential profile and a robust method for extracting it from time series of motor positions. J Theor Biol 242, 908–921 (2006). 16806275 10.1016/j.jtbi.2006.04.005

[b42] Lukic B. . Motion of a colloidal particle in an optical trap. Physical Review E 76 (2007). 10.1103/PhysRevE.76.011112 17677415

[b43] Kunwar A. & Mogilner A. Robust transport by multiple motors with nonlinear force-velocity relations and stochastic load sharing. Phys Biol 7 (2010). 10.1088/1478-3975/7/1/016012 PMC2858005 20147778

[b44] Kunwar A. . Mechanical stochastic tug-of-war models cannot explain bidirectional lipid-droplet transport. Proc Natl Acad Sci USA 108, 18960–18965 (2011). 22084076 10.1073/pnas.1107841108 PMC3223464

[b45] Soppina V., Rai A. K., Ramaiya A. J., Barak P. & Mallik R. Tug-of-war between dissimilar teams of microtubule motors regulates transport and fission of endosomes. Proc Natl Acad Sci USA 106, 19381–19386 (2009). 19864630 10.1073/pnas.0906524106 PMC2770008

[b46] Li Y., Zhang W., Niu J. & Chen Y. Mechanism of Photogenerated Reactive Oxygen Species and Correlation with the Antibacterial Properties of Engineered Metal-Oxide Nanoparticles. Acs Nano 6, 5164–5173 (2012). 22587225 10.1021/nn300934k

[b47] Zhang W., Li Y., Niu J. & Chen Y. Photogeneration of Reactive Oxygen Species on Uncoated Silver, Gold, Nickel, and Silicon Nanoparticles and Their Antibacterial Effects. Langmuir 29, 4647–4651 (2013). 23544954 10.1021/la400500t

[b48] Fang C., Bourdette D. & Banker G. Oxidative stress inhibits axonal transport: implications for neurodegenerative diseases. Mol. Neurodegener. 7 (2012). 10.1186/1750-1326-7-29 PMC3407799 22709375

[b49] Prousek J. Fenton chemistry in biology and medicine. Pure Appl Chem 79, 2325–2338 (2007).

[b50] Pereira M. C., Oliveira L. C. A. & Murad E. Iron oxide catalysts: Fenton and Fenton-like reactions - a review. Clay Miner. 47, 285–302 (2012).

[b51] Malvindi M. A. . Toxicity Assessment of Silica Coated Iron Oxide Nanoparticles and Biocompatibility Improvement by Surface Engineering. Plos One 9 (2014). 10.1371/journal.pone.0085835 PMC3897540 24465736

[b52] Chen O. . Magneto-fluorescent core-shell supernanoparticles. Nat. Commun. 5 (2014). 10.1038/ncomms6093 PMC4264679 25298155

[b53] Fonseca S. M. . Triplet-state and singlet oxygen formation in fluorene-based alternating copolymers. J. Phys. Chem. B 110, 8278–8283 (2006). 16623508 10.1021/jp060251f

[b54] Pu K., Shuhendler A. J. & Rao J. Semiconducting Polymer Nanoprobe for *In Vivo* Imaging of Reactive Oxygen and Nitrogen Species. Angewandte Chemie-International Edition 52, 10325–10329 (2013). 10.1002/anie.201303420 PMC4079533 23943508

[b55] Pu K. . Semiconducting polymer nanoparticles as photoacoustic molecular imaging probes in living mice. Nat. Nanotechnol. 9, 233–239 (2014). 24463363 10.1038/nnano.2013.302 PMC3947658

[b56] Hoffman-Kim D., Diefenbach T. J., Eustace B. K. & Jay D. G. in *L*aser *Man*ipu*la*tion *of Cells and Tissues* Vol. 82 Methods in Cell Biology (eds Berns M. W. & Greulich K. O.) 335–354 (2007). 17586263 10.1016/S0091-679X(06)82011-X

[b57] Jacobson K., Rajfur Z., Vitriol E. & Hahn K. Chromophore-assisted laser inactivation in cell biology. Trends Cell Biol 18, 443–450 (2008). 18706812 10.1016/j.tcb.2008.07.001 PMC4445427

[b58] Beck S. . Fluorophore-assisted light inactivation: A high-throughput tool for direct target validation of proteins. Proteomics 2, 247–255 (2002). 11921440 10.1002/1615-9861(200203)2:3<247::aid-prot247>3.0.co;2-k

[b59] Bruno L., Echarte M. M. & Levi V. Exchange of Microtubule Molecular Motors During Melanosome Transport in Xenopus laevis Melanophores is Triggered by Collisions with Intracellular Obstacles. Cell Biochem Biophys 52, 191–201 (2008). 19002657 10.1007/s12013-008-9034-3

[b60] Zajac A. L., Goldman Y. E., Holzbaur E. L. F. & Ostap E. M. Local Cytoskeletal and Organelle Interactions Impact Molecular-Motor-Driven Early Endosomal Trafficking. Curr Biol 23, 1173–1180 (2013). 23770188 10.1016/j.cub.2013.05.015 PMC3738301

[b61] Bruno L., Salierno M., Wetzler D. E., Desposito M. A. & Levi V. Mechanical Properties of Organelles Driven by Microtubule-Dependent Molecular Motors in Living Cells. Plos One 6 (2011). 10.1371/journal.pone.0018332 PMC3069964 21483765

