# Supplementary material for: Nanoparticle-assisted optical tethering of endosomes reveals the cooperative function of dyneins in retrograde axonal transport
Source: Sci Rep. 2015 Dec 10;5:18059. doi: 10.1038/srep18059 (PMC4674899; doi:10.1038/srep18059)
Supplement: Supplementary Information [file srep18059-s1.pdf]

Nanoparticle-assisted optical tethering of endosomes reveals the cooperative function of dyneins in retrograde axonal transport

Praveen D. Chowdary[a], Daphne L. Che[a], Luke Kaplan[a], Ou Chen[b], Kanyi Pu[c], Moungi Bawendi[b] and Bianxiao Cui[a,*]

[a] Department of Chemistry, Stanford University, 380 Roth Way, Stanford, CA 94305, USA;

[b] Department of Chemistry, Massachussets Institute of Technology, 77 Massachussets Ave, Cambridge, MA 02139, USA; [c] School of Chemical and Biomedical Engineering, Nanyang Technological University, 62 Nanyang Drive, N1.3, B2-05, Singapore 637459

Supplementary Information: Table of Contents

1. Experiments and data processing

1.1 Materials

1.2 Conjugation of commercial iron oxide nanoparticles with WGA and fluorophores

1.3 Synthesis of biotinylated silica-coated iron oxide nanoparticles and WGA conjugation

1.4 Synthesis of biotinylated polymer nanoparticles and WGA conjugation

1.5 Oblique illumination imaging of axonal transport in microfluidic DRG neuron culture

1.6 Single particle tracking of endosome transport in time-lapse movies

1.7 Kymograph processing to extract the endosome jumps from time-lapse movies

2. Detachment model and fitting the endosome jumps

2.1 Solutions for the detachment model in Equation (2) of main text

2.2 Fitting the endosome jump to the motor-detachment model

3. Supplementary data

3.1 Quantification of retrograde transport velocities from endosome trajectories

3.2 Occasional jumps observed with QD-endosomes and DiI-endosomes in axons

3.2 Analysis of endosome deformation during jumps

3.3 Considerations of nanoparticle heating under continuous laser illumination

1. Experiments and data processing

1.1 Materials:

The chemicals used in this work and their sources are listed here. QD655-WGA, Alexa555-WGA, DiI (Invitrogen). Biotin-WGA (Vector Labs). Streptavidin, F8BT (Sigma Aldrich), DPSE-PEG-Biotin (Avanti Polar Lipids).

1.2 Conjugation of commerical iron oxide nanoparticles (INPs) with WGA and fluorophores:

Single core superparamagnetic iron oxide nanoparticles (INPs, 30±2.5nm, 50±5nm) with very low size tolerances were purchased from Ocean Nanotech. These INPs, with carboxylic acid surface functionalization, were coated with fluorescent Alexa555-WGA by standard carbodiimide chemistry to obtain fluorescent INP30-WGA and INP50-WGA. This reaction was carried out at 1:1000 ratio of INP:WGA, and multiple magnetic separations were done in a high gradient magnetic separator (SuperMag, Ocean Nanotech) to discard unconjugated WGA.

Multicore iron oxide nanoparticles (BNF-Starch-redF, 100nm, intensely fluorescent), custom conjugated with fluorescent dye DY-555 and streptavidin functionalization, were purchased from Micromod. These particles had a mean diameter of 107nm and a polydispersity index < 0.2 reported by Micromod using photo correlation spectroscopy. We conjugated these particles with biotin-WGA (1:60 ratio, 20min at 37C) to obtain fluorescent INP100-WGA.

1.3 Synthesis of biotinylated silica-coated iron oxide particles and WGA conjugation:

Silica-coated core-shell nanoparticles of tunable size, with multiple iron oxide cores and quantum dot shells were synthesized as described earlier[1]. Briefly, 1 mL chloroform solution containing QDs (655nm emission, 9.0 ± 0.4 nm) and INPs (5.9 ± 0.3 nm) was injected into 1 mL DTAB aqueous solution (20 mg/mL in Nanopure water). The solution was thoroughly mixed by vortexing for 5 second. After removing the chloroform from the mixture by blowing Ar at room temperature, a clear QD-INP-micelle aqueous solution was obtained. This QD-INP-micelle solution was swiftly injected into 5mL poly(vinylpyrrolidone) (PVP, MW 55000) ethylene glycol (EG) solution (2 mM) under vigorous stirring (700 rpm for 30 min) at room temperature. 2 mg of the resulting core-shell superparticles (CS-SPs) were isolated by centrifugation and redispersed in 20 mL ethanol. Under vigorous stirring, 3 mL deionized-water (DI-water) and 1 mL $NH_3{\cdot}H_2O$ solution was added dropwise into the reaction solution, followed by the addition of 50 µL TEOS. The final solution was kept stirring at room temperature for 20 min. The resulting silica-coated CS-SPs were isolated by centrifugation, washed three times with DI-water and finally dissolved in 2 mL DI-water.

Biotinylation: The silica-coated CS-SPs in DI-water (2 mL) were diluted in 10 mL DI-water. Under magnetic stirring, 50 µL $NH_3{\cdot}H_2O$ solution and 20 µL TEOS were added into the solution, sequentially. After the mixture solution was heated to 70 °C, 1 mL Biotin-PEG-silane (MW 5000) aqueous solution (0.02 M) was dropped in. The reaction solution was kept stirring at 70 °C for 3 hrs and then stirred at room temperature for another 15 hrs. The resulting biotinylated silica-

coated CS-SPs were isolated by centrifugation, washed three times with nanopure water and finally dissolved in 1 mL phosphate buffer solution (PBS, 1X) and stored at ~4 °C. Figure S1a shows the dynamic light scattering measurement of the silica-coated nanoparticles, which gave an average hydrodynamic size of 164nm. We functionalized these particles with streptavidin using a stoichiometric amount of streptavidin and subsequently conjugated with biotin-WGA (1:300 ratio) for the axonal transport studies.

1.4 Synthesis of biotinylated polymer nanoparticles (b-PN) and WGA conjugation:

The b-PNs are synthesized according to previously published procedures[2-4]. Briefly, a mixed THF solution (1 mL) containing poly(9,9-dioctylfluorene-alt-benzothiadiazole) (F8BT) (0.25 mg/mL) and 1,2-distearoyl-sn-glycero-3-phosphoethanolamine-*N*-[biotinyl(poly(ethylene glycol))-2000] (ammonium salt) (DPSE-PEG-Biotin) (2.5 mg/mL) was used to prepare b-PNs by rapidly injecting it into distilled-deionized water (9 mL) under continuous sonication with a microtip-equipped probe sonicator. After sonication, THF was evaporated under nitrogen atmosphere. The aqueous solution was filtered through a polyethersulfone (PES) syringe driven filter (0.22 µm, Millipore), and washed three times using a 30K centrifugal filter unit (Millipore). The nanoparticle solution was finally concentrated to 0.08 mg/mL (based on the mass of PN) by ultrafiltration and stored in dark at 4 °C. Figure S1b shows the absorption and emission of the b-PNs. Figure S1c shows the dynamic light scattering measurement of the b-PNs, which showed an average hydrodynamic size of 31.3nm with a polydispersity of 0.176. We functionalized the b-SPNs with streptavidin using a stoichiometric amount of streptavidin and conjugated with biotin-WGA (1:5 ratio) to obtain fluorescent PN-WGA for axonal transport studies.

1.5 Oblique illumination imaging of axonal transport in microfluidic DRG neuron culture:

The use of microfluidic devices for primary neuron culture and imaging axonal transport had been documented by us elsewhere[5,6]. In this work, we used mature DRG neuron cultures (7-10 days old) for the QD-WGA and INP-WGA transport studies.

An inverted microscope (Nikon Eclipse Ti-U), customized with dual laser (488, 561nm) laser excitation and three-color imaging, was set for oblique illumination. Briefly, the laser beam was expanded to 3 cm diameter and focused at the back focal plane of the microscope objective (CFI Plan Apo TIRF, 100X/1.49NA) for a collimated illumination with beam waist ~120µm. Starting from total internal reflection geometry, the incident angle was gradually lowered below the critical angle till the QD-endosomes within the axons are made visible by the oblique angle illumination. Since the microfluidic channels in our device are 3 µm high, a penetration depth of 1-3 µm is sufficient to illuminate axonal fluorophores in multiple focal planes. The fluorescence collected by the objective was spectrally split into three different bands by two sets of dichroics. These bands were relayed and focused onto a third of an EMCCD sensor (Andor Ixon DU-897).

The axon terminals in the mcirofluidic culture are incubated with Alexa-WGA (0.5nM) or QD-WGA (0.5 nM) or INP-WGA (1-2nM) for 45 min, which was then washed off by the culture medium. Shortly before imaging, the culture medium was replaced by CO_2 independent medium and the culture was imaged on a water-heated custom microscope stage set to maintain the culture at 37 ºC. Imaging was started typically 2 hrs after the incubation start and restricted to <

45 min session. Time-lapse movies of endosome transport were acquired at 10-150 frames per second and the imaging laser power is varied in the range 0.13-11 mW (i.e. 1.4-114 W/cm^2, for a beam waist ~120 μm) using neutral density filters. For imaging DiI stained retrograde endosomes in axons, we incubated the axon terminals with ~2μM DiI for 10min, and the imaging was done 1.5hrs after incubation as described above.

1.6 Single particle tracking of the endosome transport in time-lapse movies:

Semi-automated custom Matlab software is used to extract endosome trajectories *(X(t),Y(t))* from the time lapse movies of axonal transport as described earlier[7]. Briefly, we used 2D-Gaussian fitting adapted to detect the locations of endosomes (with Alexa/QD/INP) in each movie frame. Intensity and signal-to-noise ratio cutoffs were adjusted to achieve localization accuracy < 20 nm. We then used a particle-tracking algorithm, adapted from Jaqaman et al.[8], to link these detected locations into endosome trajectories. The flux of endosome transport, kept sparse by controlled incubation conditions, lead to minimal tracking errors. Occasional errors associated with crossing trajectories are corrected by manual inspection in the final step of processing. For each endosome trajectory, we extracted the underlying microtubule track (Q^x,Q^y) using an edge-based tracing algorithm. Using this microtubule track, the endosome trajectory is converted from camera pixels *(X(t),Y(t))* to microtubule coordinates $(Q^{||}(t),Q^{\perp}(t))$ representing the motion parallel and perpendicular to the microtubule[7]. Since the microfluidic channels (and hence the axons) are aligned along the Y-axis of the camera, *Y(t)* is an approximation for $Q^{||}(t)$.

1.7 Kymograph processing to extract the endosome jumps from time-lapse movies:

Considering the lower signal-to-noise ratio of the INP-WGA endosomes imaged at 150 fps, we used a semi-automated kymograph-based analysis to accurately capture the INP-endosome detachments (Fig. S2). First, we projected the intensities of all frames in the time-lapse movie to obtain a time-projection image, which gives the diffraction limited intensity profiles of the microtubular tracks of INP-WGA endosomes in axons (Fig. S2b). Then we manually traced the centerline of each intensity profile, which is refined by an automated centerline tracer, to give the underlying microtubule track *Q(x,y)* (red curve in Fig. S2b). We then generated the kymograph *I(Q,t)*, which depicts the intensity variation along the microtubular track *Q* as a function of timeframe, due to the motion of INP-WGA endosomes (Fig. S2c). We fit the intensity profile at each timeframe *I(Q)* with a 1D Gaussian mixture model to obtain the INP-WGA endosome locations along the microtubular track at each time frame (Fig. S2d). These locations are then connected by 1D single particle tracking to obtain the INP-WGA endosome motion parallel to the microtubule i.e. $Q^{||}(t)$ (Fig. S2e).

The position uncertainties in $Q^{||}(t)$ (Fig. S2e, parenthesis) are computed from the integrated intensities of Gaussian fits using the calibration equation (Fig. 3b of main text). The calibration equation is obtained from the localization accuracy of INPs (with a wide range of intensities) stuck on coverslips as follows. We tracked the INP positions for over 3s (>400 frames) using the kymograph analysis described above. The standard deviation in the NP positions represented the localization inaccuracy in our system (imaging and processing). Figure 3b in main text shows the

localization accuracy as a function of the integrated intensity of the INPs and the approximate power-law calibration curve. We selected the intensity cutoff to achieve < 25nm accuracy.

2. Detachment model and fitting the endosome jumps

2.1 Solutions for the detachment model in Equation (2) of main text:

Ignoring the fast inertial and Brownian fluctuations compared to the motor driven endosome motion, Equation (1) in the main text simplifies to Equation (S1) (Equation (2) in main text).

$$\frac{dq}{dt} = \frac{F_{motor}}{\gamma} - \frac{k(q-q_0)}{\gamma} \qquad \text{(S1)}$$

Pre-detachment solution: The stochastic load sharing among dyneins leads to complex pre-detachment stall profiles of endosomes under load that cannot be described analytically. Under mean field assumption, where the load is shared equally by all dyneins, we approximated the average endosome velocity by a simple linear force-velocity relation in Equation (S2).

$$\frac{dq}{dt} = v_m\left(1 - \frac{k(q-q_0)}{nF_s}\right) \qquad \text{(S2)}$$

where v_m is the unloaded velocity of the endosome in Stokes regime, k is the stiffness of the elastic tether, $(q\text{-}q_0)$ is the stretch of the tether, nF_s is the cumulative stall force of *'n'* dyneins (with unitary stall force F_s) moving the endosome. The solution of S2 is given by Equation (S3), which is only applicable under the mean field assumption.

$$q(t) = q_0 + \frac{A}{B}\left(1 - \exp\left(-B(t-t_0)\right)\right); \quad A = v_m; \quad B = \frac{v_m k}{nF_s}; \; t < t_d \qquad \text{(S3)}$$

Post-detachment solution: Equation (S1) simplifies considerably after the detachment of leading-dyneins from the microtubule with $F_{motor}=0$. The endosome recoil in the medium with friction coefficient γ is described by the solution given in Equation (S4), where V_{detach} is the instantaneous recoil velocity of the endosome at detachment, and k/γ is the damping constant of endosome recoil.

$$q(t) = q_s + \frac{C}{D}\left(1 - \exp\left(-D(t-t_d)\right)\right); \quad C = V_{detach}; \quad D = \frac{k}{\gamma}; \; t > t_d \qquad \text{(S4)}$$

2.2 Fitting the endosome jump to the motor-detachment model:

The primary objective of fitting an endosome jump is to obtain the instantaneous detachment velocity V_{detach}, which is proportional to the cumulative stall force of leading-dyneins. In principle, V_{detach} can be obtained by fitting the post-detachment recoil profile to Equation (S4). However, it is important to accurately identify the detachment time (t_d) and position (q_s) for

accurate extraction of V_{detach} by model fitting (see Fig. 3a in main text). Fitting the pre-detachment stall and post-detachment recoil profiles simultaneously, provides reliable estimation of (t_d, q_s) as the intersection point of the fit curves.

Though Eq. S3 is not applicable to stall profiles of dyneins with stochastic load sharing, it is useful in fitting the gradually stalling segment prior to detachment as in Fig. 3c of main text. However, the parameters A and B obtained by fitting Eq. S3 to a partial range of stall profiles are not relevant for the interpretation of v_m (unloaded dynein velocity), k (tether stiffness) and F_s (unitary dynein stall force), as given in Equation (S3). In this analysis, Equation (S3) merely serves as a smoothly varying polynomial that aids in the objective estimation of (t_d, x_s) in conjunction with Equation (S4), which is used to fit the post-detachment recoil profile.

We fit each of the INP100-endosome jumps to Equations (S3), (S4) by χ^2 minimization. The global fitting of the pre-detachment and post detachment curves weighted by the position uncertainties in INP100-endosome position yields t_d, x_s, V_{detach} and k/γ. In many cases the endosome recoil showed sudden deviations from exponentiality within 40-70ms of detachment, presumably due to fast dynein rebinding or collisions with local cytoskeletal features. Therefore, we typically fit ~40-70ms (4-10 time points) of the post-detachment recoil profile depending on the jump profile. Figure S3 shows a random selection of a few INP100-endosome jumps and the model fits.

The damping parameter k/γ is expected to be constant for a given tethered endosome exhibiting jumps within a narrow stretch of the axon. However, as shown for the endosome in Fig. 3 of the main text, we notice a dispersion of k/γ values even for the same endosome due to A) the deviations from exponentiality in jumps and B) the standard errors in fitting a limited range of data (4-10 time points) within localization uncertainties. In order to mitigate these factors and improve the accuracy of the fit parameters (V_{detach} and k/γ) we only selected the jumps that resulted in minimal standard errors (<15% of the fit parameter) in fitting V_{detach}. These constituted 32% of the total number of jumps identified in our data (306 out of 961 jumps).

3. Supplementary data

3.1 Quantification of retrograde transport velocities from endosome trajectories

We used a Bayesian parsing approach reported earlier[7] to identify the retrograde moving, pausing, and anterograde moving phases within each endosome trajectory (>10sec duration). Briefly, we parsed each endosome trajectory (10fps imaging, $Q^{||}(t)$ obtained by particle tracking and processing described in 1.6) into a series of constant velocity segments (minimum length 0.3s imposed). We then clubbed consecutive segments in the same direction to obtain retrograde runs, pauses, and anterograde runs. A run is a sustained period of motion (> 0.5s duration, ≥ 0.2 μm/s velocity) in the same direction that ends with a pause or a direction reversal. A pause is defined as a motionless segment > 0.5s duration with < 0.2 μm/s velocity.

In order to analyze the retrograde run velocity distribution, we acquired a large dataset of QD-WGA endosome trajectories (10fps imaging, N=1141). Figure S4a shows the distribution of the average speed of QD-WGA endosomes including pauses and direction reversals in motion.

Figure S4b shows the distribution of retrograde run velocities of the QD-WGA endosomes. We do not notice any obvious multimodal pattern that is indicative of the number of dyneins on the endosomes.

3.2 Occasional jumps observed with retrograde QD-endosomes and DiI-endosomes in axons:

The frequency of endosome jumps/stalling showed a dramatic increase with laser power in the case of INP-endosomes. The endosome jumps were also occasionally exhibited by retrograde QD-WGA-endosomes and DiI-stained retrograde endosomes (Fig. S5, Movie S5, Movie S4). However, we do not notice any laser power dependence in the range of 1.4-114 W/cm^2. Further, many QD- and DiI-endosomes resumed the retrograde motion after exhibiting a series of jumps (Movie S5). The characteristics of stall profiles and jumps are similar to that of INP-endosomes.

3.3 Analysis of endosome deformation during jumps:

It had been shown that endosomes of size > 500 nm stretched ~25% of their size during tugs-of-war between dyneins and kinesins[9]. Axonal endosomes (50-250 nm) are expected to be more rigid due to their smaller size. In order to quantify the extent of endosome stretching at stall (i.e F_{opp}=$F_{dyneins}$), we analyzed the jumps of DiI-stained fast retrograde endosomes. We found that DiI endosomes remained nearly diffraction limited and showed minimal deformation during jumps. Figure S6a shows the kymograph of DiI endosome jumps of ~1µm size. The peak endosome intensity and the Gaussian width (HWHM~350nm) remained nearly constant during the jump (Fig. S6c). Note that the HWHM is not an accurate metric for the absolute vesicle size but is a useful qualitative measure to assess the vesicle deformation at varying loads. On average the HWHM of DiI endosomes increased by < 7% from baseline to stall (Fig. S6e) and this increase contributed < 4% to the endosome jump size (Fig. S6f). This indicates that endosome deformation is contributing minimally to the elastic stretch responsible for the jumps in NOTE.

3.4 Considerations of nanoparticle heating under continuous laser illumination:

The role of local temperature spikes, due to the photothermal response of INPs, in causing the endosome jumps can be ruled out due to the following reasons. First, the local heating of an isolated INP by continuous laser excitation is negligible due to rapid diffusion of heat (<100ns) in the medium[10]. The steady state surface temperature of a 50 nm INP in water is estimated to be < 0.1K higher than that of the medium (see below). Second, the dynein stepping rate would be sensitive to INP heating[5,7] by virtue of rapid heat diffusion and the distance (<100nm) between the INP-endosome and the dynein head domains. However, we did not see any increase in endosome speed corresponding to endosome jumps, which suggests that the local temperature changes are negligible.

The thermal relaxation for a spherical particle with radius R is given by $\tau = R^2/D$, where D is the thermal diffusivity of the medium (10^{-7}m^2/s for water)[10]. For a particle of size 10-100nm the relaxation is fast and within 1-100ns. The steady state temperature difference at a particle surface upon continuous laser illumination is given by $dT = \sigma_{abs} I_0/(4\pi\kappa R)$, where σ_{abs} is the absorption cross section of the particle, I_0 is the laser irradiance, and κ the thermal conductivity of the

medium[10,11]. Given a maximum possible absorption cross section of 1e-14m^2 for an iron oxide nanoparticle (R=50nm), and the maximum laser irradiance tried in our imaging system (1150kW/m^2), we estimate dT~0.03K.

References:

1 Chen, O. *et al.* Magneto-fluorescent core-shell supernanoparticles. *Nat. Commun.* **5**, (2014).

2 Pu, K. *et al.* Phosphorylcholine-Coated Semiconducting Polymer Nanoparticles as Rapid and Efficient Labeling Agents for In Vivo Cell Tracking. *Advanced Healthcare Materials* **3**, 1292-1298, (2014).

3 Pu, K., Shuhendler, A. J. & Rao, J. Semiconducting Polymer Nanoprobe for In Vivo Imaging of Reactive Oxygen and Nitrogen Species. *Angewandte Chemie International Edition* **52**, 10325-10329, (2013).

4 Pu, K. *et al.* Semiconducting polymer nanoparticles as photoacoustic molecular imaging probes in living mice. *Nat Nano* **9**, 233-239, (2014).

5 Zhang, K. *et al.* Single-molecule imaging of NGF axonal transport in microfluidic devices. *Lab Chip* **10**, (2010).

6 Cui, B. X. *et al.* One at a time, live tracking of NGF axonal transport using quantum dots. *Proc Natl Acad Sci U S A* **104**, 13666-13671, (2007).

7 Chowdary, P. D., Che, D. L., Zhang, K. & Cui, B. Retrograde axonal NGF transport - Motor coordination in the unidirectional motility regime. *Biophys J* **108**, 2691-2703, (2015).

8 Jaqaman, K. *et al.* Robust single-particle tracking in live-cell time-lapse sequences. *Nat Meth* **5**, 695-702, (2008).

9 Soppina, V., Rai, A. K., Ramaiya, A. J., Barak, P. & Mallik, R. Tug-of-war between dissimilar teams of microtubule motors regulates transport and fission of endosomes. *Proc Natl Acad Sci U S A* **106**, 19381-19386, (2009).

10 Keblinski, P., Cahill, D. G., Bodapati, A., Sullivan, C. R. & Taton, T. A. Limits of localized heating by electromagnetically excited nanoparticles. *J. Appl. Phys.* **100**, (2006).

11 Baffou, G. *et al.* Photoinduced Heating of Nanoparticle Arrays. *Acs Nano* **7**, 6478-6488, (2013).

Supplementary Information: Figures

Figure S1:

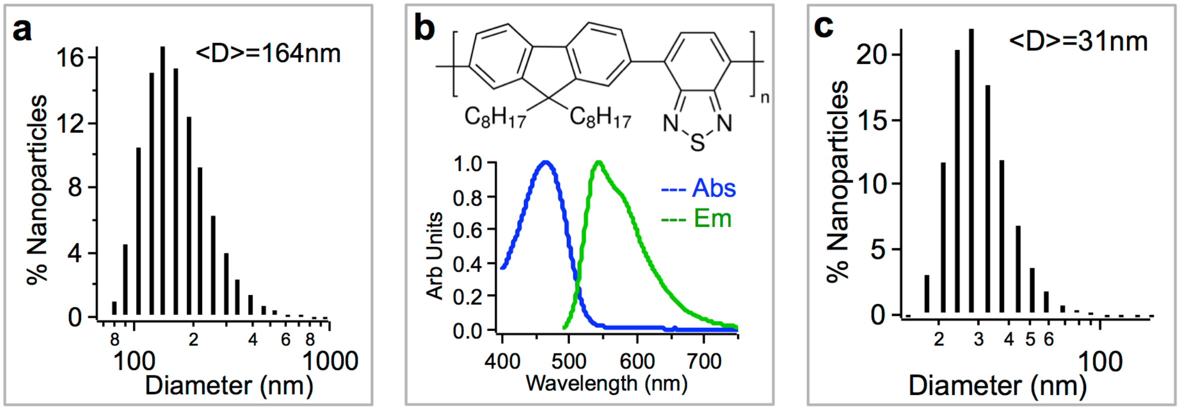

Figure S1: Silica-coated iron oxide and polymer nanoparticles. a) Dynamic light scattering measurement of silica-coated INPs showing a mean size of 164nm. b) Structure of the polymer (F8BT) within the polymer nanoparticles and the absorption/emission spectra of the particles. c) Dynamic light scattering of polymer nanoparticles shows a mean size of 31nm with a polydispersity of 0.176.

Figure S2:

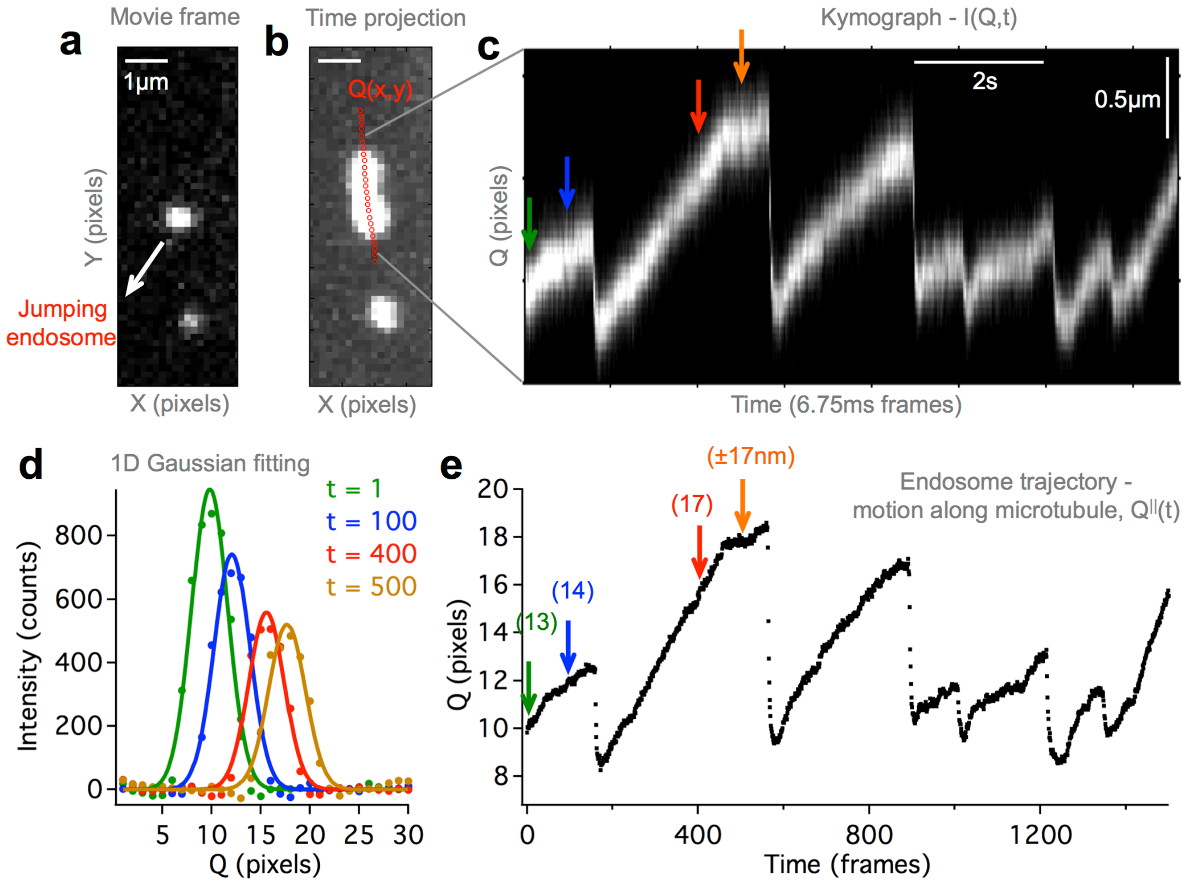

Figure S2: Kymograph based processing of INP-endosome motion (Description in section 1.7). a) Movie frame from the time-lapse movie (Movie S6) of INP100-endosome jumps in Fig. 3c. b) Time projection image of the pixel intensities in the movie showing the diffraction limited tracks of INP100-endosomes. The centerline of the intensity profile (in red) approximates the underlying microtubule track *Q(x,y)*. c) Kymograph *I(Q,t)* depicting the intensity variation along *Q(x,y)* as a function of the movie frame. d) 1D Gaussian fits of the intensity profile along *Q(x,y)* at specified timeframes to identify the position of the INP-endosome. e) INP-endosome position along the microtubule as a function of time $Q^{//}(t)$. Uncertainties (nm) in INP-endosome positions, computed from the integrated intensities of the Gaussian fits, are shown in parenthesis.

Figure S3:

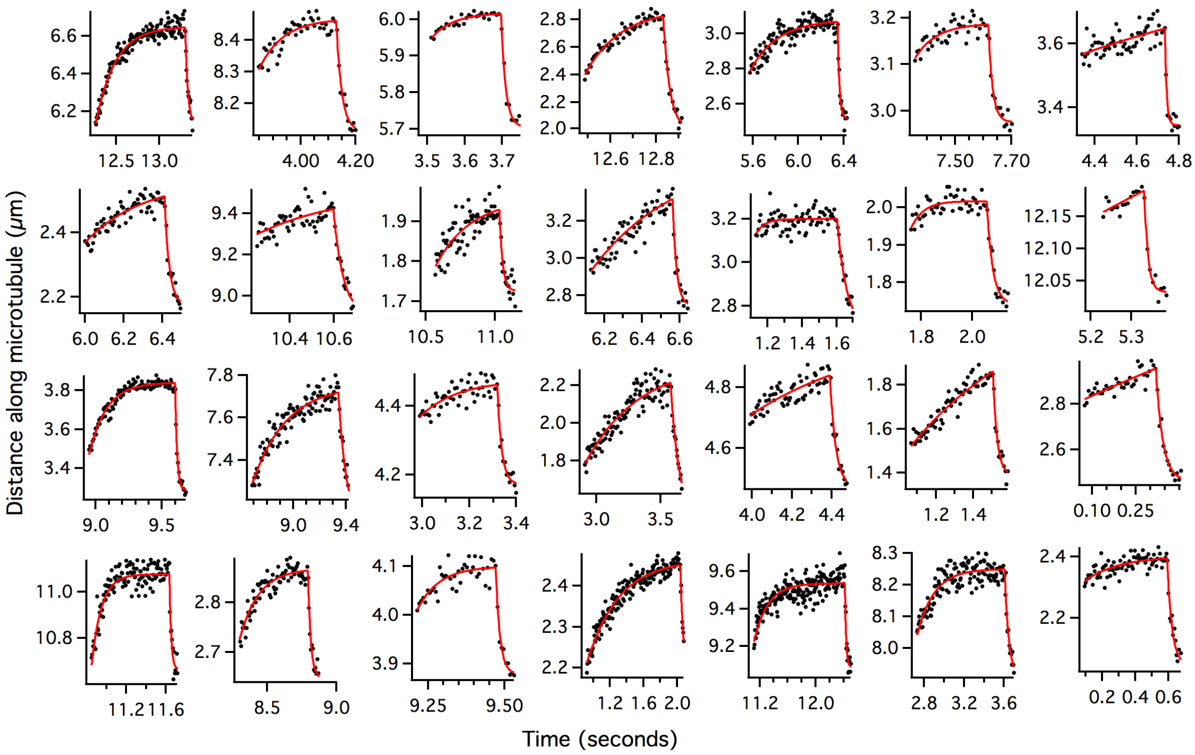

Figure S3: Random selection of a few INP100-endosome jumps and model fits (Description in section 2.2).

Figure S4:

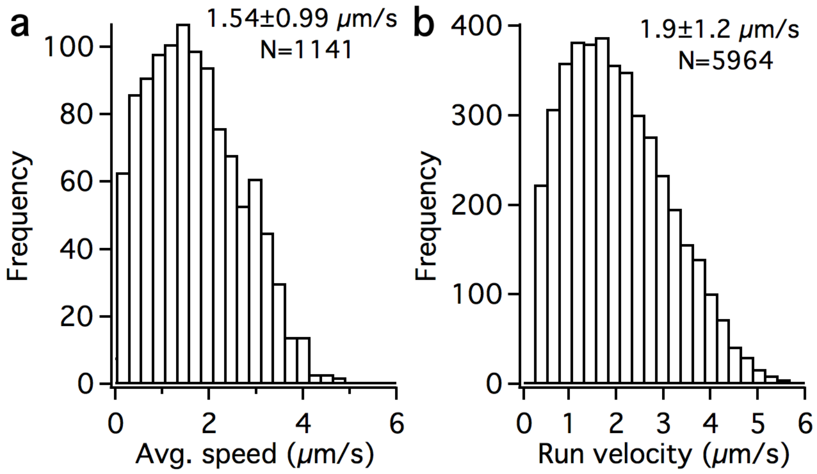

Figure S4: Quantification of retrograde run velocities from endosome trajectories (Description in section 3.1). a) Average speed of retrograde QG-WGA endosomes (N=1141 endosomes). b) Retrograde run velocities of QD-WGA endosomes (N=5964 runs).

Figure S5:

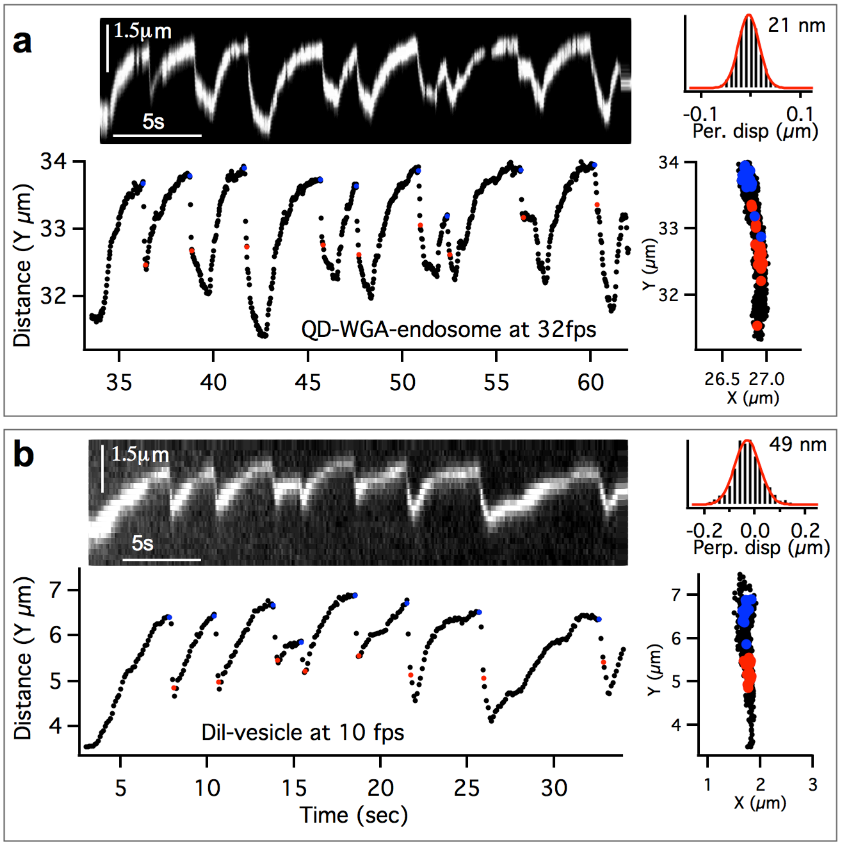

Figure S5: Occasional jumps seen with retrograde a) QD-WGA-endosomes (32fps, Movie S3) and b) DiI-stained endosomes (10fps, Movie S4) in axons. Within each panel we show the kymograph (top left), the approximate motion along the microtubule, $Y(t) \sim Q^{//}(t)$ (bottom left), the microtubule track depicted by tracked cargo positions X,Y (bottom right), and the histogram of displacement perpendicular to the microtubule (top right). Endosome positions at stall and a few timeframes (125ms for QD, 300ms for DiI) after detachment are shown in blue and red respectively in the bottom panels.

Figure S6:

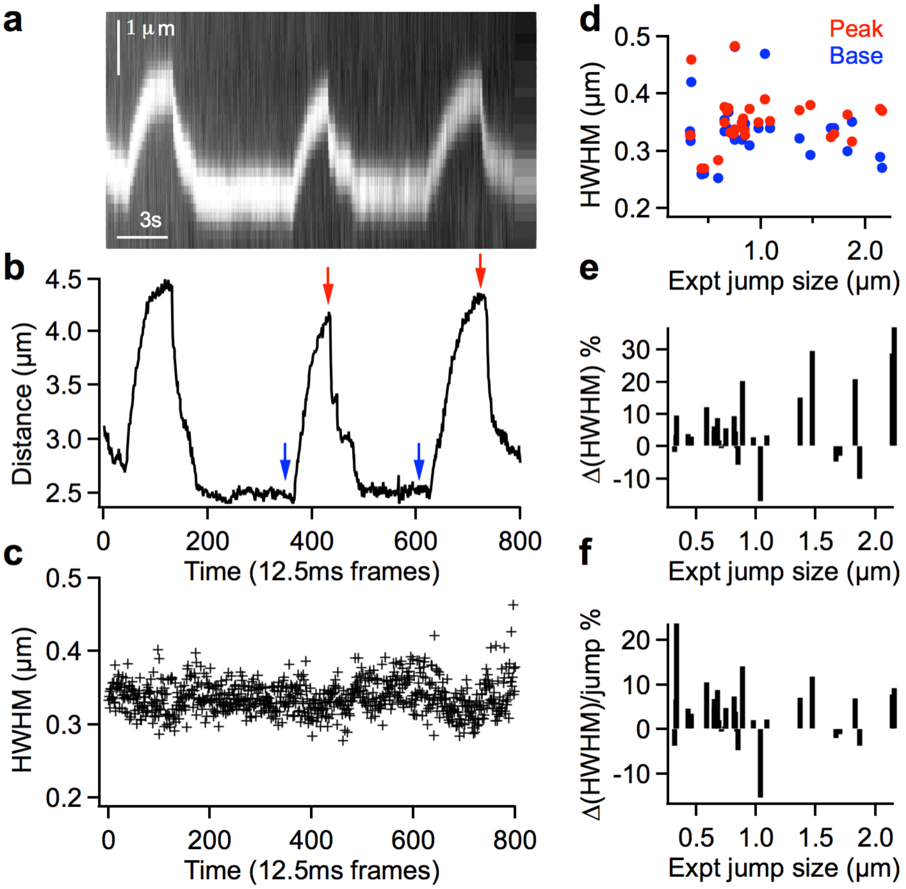

Figure S6: Qualitative assessment of endosome deformation during jumps (Description in section 3.3). a) Kymograph of retrograde DiI-stained endosome jumps (80fps). 1D Gaussian fits to the intensity profile at each time frame gave the vesicle position shown in (b) and the half width at half maximum (HWHM) values shown in (c). The HWHM, which is a qualitative measure of the endosome stretching (beyond diffraction limit) along microtubule, remained nearly constant during the jumps. d) The HWHM of jumps computed at the baseline (blue arrows in (b) and the stall position (red arrows in b) of jumps. e) 74% of jumps (N=31) showed an increase (average < 7%) in HWHM from baseline to stall position, which is shown as a function of the jump size. f) Relative change in HWHM as a function of jump size shows that endosome stretching contributes <4% on average to the jump size.
